# Minimum Incidence of Adult Invasive Pneumococcal Disease in Blantyre, Malawi an Urban African Setting: A Hospital Based Prospective Cohort Study

**DOI:** 10.1371/journal.pone.0128738

**Published:** 2015-06-03

**Authors:** Naor Bar-Zeev, Neema Mtunthama, Stephen B Gordon, Gershom Mwafulirwa, Neil French

**Affiliations:** 1 Malawi-Liverpool-Wellcome Trust Clinical Research Programme, College of Medicine, University of Malawi, Blantyre, Malawi; 2 Institute of Infection and Global Health, University of Liverpool, Liverpool, United Kingdom; 3 Department of Clinical Sciences, Liverpool School of Tropical Medicine, Liverpool, United Kingdom; University of Cambridge, UNITED KINGDOM

## Abstract

Invasive pneumococcal disease causes substantial morbidity and mortality in Africa. Evaluating population level indirect impact on adult disease of pneumococcal conjugate vaccine (PCV) programmes in infants requires baseline population incidence rates but these are often lacking in areas with limited disease surveillance. We used hospital based blood culture and cerebrospinal fluid surveillance to calculate minimal incidence of invasive pneumococcal disease in the adult (≥15 years old) population of Blantyre, a rapidly growing urban centre in southern Malawi, in the period preceding vaccine introduction. Invasive pneumococcal disease incidence in Blantyre district was high, mean 58.1 (95% confidence interval (CI): 53.7, 62.7) per 100,000 person years and peaking among 35 to 40 year olds at 108.8 (95%CI: 89.0, 131.7) mirroring the population age prevalence of HIV infection. For pneumococcal bacteraemia in urban Blantyre, mean incidence was 60.6 (95% CI: 55.2, 66.5) per 100,000 person years, peaking among 35 to 40 year olds at 114.8 (95%CI: 90.3, 143.9). We suspected that our surveillance may under-ascertain the true burden of disease, so we used location data from bacteraemic subjects and projected population estimates to calculate local sub-district incidence, then examined the impact of community level socio-demographic covariates as possible predictors of local sub-district incidence of pneumococcal and non-pneumococcal pathogenic bacteraemia. Geographic heterogeneity in incidence was marked with localised hotspots but ward level covariates apart from prison were not associated with pneumococcal bacteraemia incidence. Modelling suggests that the current sentinel surveillance system under-ascertains the true burden of disease. We outline a number of challenges to surveillance for pneumococcal disease in our low-resource setting. Subsequent surveillance in the vaccine era will have to account for geographic heterogeneity when evaluating population level indirect impact of PCV13 introduction to the childhood immunisation program.

## Introduction

*Streptococcus pneumoniae* is an important pathogen particularly in children and HIV infected adults in sub-Saharan Africa[1–3]. Pneumococcal conjugate vaccination of children has resulted in reduced invasive pneumococcal disease (IPD) incidence in unvaccinated adults in industrialised countries and in HIV-infected adults in middle income countries [2,4–6]. A similar effect from paediatric vaccination in low income countries in Africa with generalised HIV epidemics would be of major benefit and obviate the need to vaccinate high risk adults in whom direct vaccination is of proven benefit[1]. However, in communities with a high burden of pneumococcal disease, diverse pneumococcal serotype distribution in invasion and carriage and high prevalence of HIV infection it is plausible that older unvaccinated children or adults may themselves be a source for ongoing community transmission of pneumococcus. It is therefore important to determine whether such indirect effects will occur with conjugate vaccine introduction and if they do whether their magnitude obviate the benefit of direct vaccination of adults. One method of determining vaccine impact is to compare pre vaccine introduction disease rates with those after introduction. Whilst conceptually simple, this method has several potential sources of error, but as minimum requirements, baseline calculation of rates of disease and consistent methods used for disease surveillance and ascertainment over time are essential.

Incidence rates for IPD are difficult to obtain in many resource poor settings because of lack of laboratory capacity or lack of epidemiological surveillance infrastructure[7]. Even where laboratory capacity is available in a sentinel site, the accurate determination of disease incidence can be challenging. Case ascertainment is hampered by prior use of antibiotics on empirical grounds which reduces the yield of a blood culture surveillance system, and this practice is common in a setting where syndromic management is encouraged[8]. Blood culturing technique may also be sub-optimal, in particular the volume of cultured blood is a key determinant of blood culture sensitivity to detect pneumococcal blood stream infection[9]. Additionally, determining the true denominator of the population can be challenging in a complex urban setting where hospitals serve both referral level and primary healthcare functions to the urban and peri-urban population, particularly when primary health facility service provision may be patchy. As a consequence very little is known about population incidence of pneumococcal disease in Africa. Few paediatric incidence estimates have been published[2,10–12], in HIV infected adult cohorts rates of over 200 per 100,000 are reported from South Africa[3] and higher still in Kenya[13], but general population incidence is lacking for low-income countries in sub-Saharan Africa. We sought to fill this gap by estimating a minimum incidence for IPD in our setting and by using available datasets to examine to what extent we may be under-ascertaining the true burden of disease.

Previous work in Blantyre Malawi had confirmed the important role of IPD as a cause of morbidity and mortality in the adult population, particularly those HIV-infected[14]. We had also shown the need to standardise blood culture surveillance in order to maximise IPD diagnosis[9]. As a component of this work, we conducted enhanced surveillance for pneumococcal bacteraemia in 2005–2006 at the adult Accident & Emergency Department of Queen Elizabeth Central Hospital (QECH) in Blantyre, Malawi. QECH is the major referral centre for the southern region of Malawi, and services the population of Blantyre district which numbered just over 1 million persons at the 2008 census[15]. QECH provides referral and primary level care to the population in Blantyre city and its peri-urban rural areas and is the only referral level facility providing free healthcare in the district [16,17]. Most severe illness episodes in persons attending for care at local clinics are referred to QECH. Although it is unknown how many severe illness episodes do not attend for care and may die at home, those who do attend care do so at QECH. Blantyre city comprises urbanised and semi-urbanised farming areas, and is similar to urban and peri-urban settings in much of sub-Saharan Africa. We report the baseline incidence rate of IPD among adults in the era preceding introduction of pneumococcal conjugate vaccination to the child population in Malawi, and estimate the degree of possible under-ascertainment of our surveillance system by examining the apparent heterogentiy in incidence.

## Materials and Methods

### Case ascertainment

All persons 15 years or older presenting to the Accident & Emergency Department of QECH with a febrile (>37.5°C) illness, clinical pneumonia or who were otherwise seriously ill to include sepsis in the differential diagnosis, on any day of the week during the period 1 January 2005 to 31 December 2006 had blood culture performed by a specialised team of phlebotomists in accordance with standard operating procedures[9] (Table 1). Blood was transported to the laboratory and processed as previously described[9]. Blood culture contaminants were defined using standard guidelines[18]. Persons presenting with suspected meningitis had lumbar puncture performed as part of routine care. We used our laboratory database to obtain the identifiers of all persons with pneumococcus isolated in cerebrospinal fluid. Pleural taps are not performed at our institution. In order to avoid double counting when cultures of blood and cerebrospinal fluid were both positive during the same illness episode we matched this list to our bacteraemic cohort on basis of identity of all 4 of given name, family name, age in years and specimen date. We accounted for linguistic characteristics of the Chichewa language (e.g. interchange of letters R and L, or the addition of letter i at the end of a word) and accepted homophonic spellings as identical (e.g. Irene and Ireen). We allowed +/-5 days between blood culture and cerebrospinal fluid specimen collection dates. The laboratory cerebrospinal fluid dataset included persons from Blantyre district but did not specify their location of residence within the district.

**Table 1:** Prevalence of Covariates Among Adults With and Without Pneumococcal Bacteraemia, Queen Elizabeth Central Hospital, Blantyre Malawi, 1 January 2005 to 31 December 2006.

Covariate	Entire cohort	Non-pneumococcal bacteraemia	Pneumococcal bacteraemia	Culture negative	*P*-value *
	N = 8,891	N = 1622	N = 559	N = 6710	
**Female**	5,065 (57.0%)	925 (57.0%)	322 (57.6%)	3818 (56.9%)	0.75
**Mean age in yrs (SD)**	34.3 (11.6)	34.7 (11.4)	34.3 (10.5)	34.2 (11.7)	0.86
**ART**	738 (8.4%)	136 (8.4%)	41 (7.3%)	561 (8.4%)	0.40
**TB therapy**	409 (4.6%)	88 (5.4%)	29 (5.2%)	292 (4.4%)	0.36
**Antifungal therapy**	65 (0.7%)	16 (1.0%)	0	49 (0.7%)	0.19
**Fever >37.5** ^**o**^ **C** †	5,884 (66.2%)	1115 (68.7%)	456 (81.6%)	4313 (64.3%)	<0.01
**Prior antibiotics**	1,907 (21.5%)	296 (18.3%)	85 (15.2%)	1526 (22.7%)	<0.01
**Prisoner**	80 (0.9%)	9 (0.6%)	12 (2.2%)	59 (0.8%)	<0.01
**Died**	109 (1.2%)	28 (1.7%)	40 (7.2%)	41 (0.6%)	<0.01

SD = standard deviation; ART = antiretroviral therapy; TB = tuberculosis

*Comparing pneumococcal bacteraemic cohort with culture negative cohort. All by X^2^-test, except antifungal therapy by Fisher's exact test with rounding up of cells.

† at presentation

### Denominators and socio-demographic indicators

Population denominators for each sub-district ward for the observation period 2005 to 2006 were derived from population growth occurring during the inter-censal period of 1998 to 2008 by assuming the difference between the population at the two censuses is linearly distributed and deriving the mean of the projected mid-year estimates for 2005 and 2006. Since this estimate is linear it likely overestimates the population at any time point, thus underestimating incidence rates, though given the proximity to the 2008 census the degree of error is likely to be small. Socio-demographic ward level indicators from the 2008 census were used[15]. Kilometre walking distance by mapped road (excludes unsealed local tracks) was measured from the centre of each ward to QECH.

### Statistical Analysis

Recalling the challenges of obtaining true population incidence of IPD, minimum estimates of hospitalised IPD incidence with exact Poisson confidence intervals were derived by dividing the number of observed IPD (pneumococcal bacteraemia plus meningitis) cases by person years of observation for the population denominators stratified by age and multiplied by 100,000. For bacteraemic cases we had data on home location so also stratified ward-specific denominators and multiplied by 100,000. We were unable to obtain data on primary health care utilisation which may have helped us understand variability in health seeking behaviour. So we explored associations between ward level socio-demographic covariates (Table 2) and incidence of hospitalised pneumococcal bacteraemia (but not meningitis) events using multivariable negative binomial regression believing these would provide an acceptable proxy. We allowed repeat events within individuals and used Huber-White robust estimators to account for the consequent correlation between outcome events[19]. Our aim thereby was to estimate to what degree our surveillance system may under-ascertain the true burden of disease. Nested models were compared and parameter associated P-values were derived from the likelihood ratio test, forward stepwise regression was also performed by including covariates achieving *P*<0.2 on univariable analysis. We did not examine variance inflation factors for our socio-demographic covariates as we were not aiming to examine the relative contribution of each factor per se, but rather aimed to adjust total ward level incidence for differences in socio-demographic covariates thereby allowing for prediction of expected number of cases by ward. We then compared these predicted expectations against the empirically observed case numbers. In so doing we were attempting to answer the following: if un-ascertained cases are indeed occurring at a rate predicted by our models, how does this modelled rate compare with the actual rate we have empirically observed? We separately modelled for associations with pneumococcal and non-pneumococcal bacteraemia. As a sensitivity analysis we reran the analysis excluding incarcerated persons. This observational study was a post-hoc analysis conducted on a cohort of patients recruited as a consequence of hospital surveillance for bloodstream infection, so we did not undertake sample size calculations for our cohort. Analyses were performed using Stata 12.1 (Statacorp, College Station, Texas).

**Table 2:** Ward Level Predictors of Pneumococcal Bacteraemia Incidence.

Covariate*	IRR (95% CI)	*P*-value
Number of households included in census from that ward	1.00 (1.0, 1.0)	0.68
Proportion of dwellings that are permanent	2.96 (0.6, 15.2)	0.19
Proportion of dwellings that are owner occupied	0.47 (0.06, 3.6)	0.47
Proportion of dwellings that have non-grass roofing ^a^	3.01 (0.4, 20.7)	0.26
Proportion of dwellings that have burnt brick or concrete walls	2.19 (0.4,11.3)	0.35
Proportion of dwellings that have sealed floor ^b^	2.81 (0.45, 17.3)	0.27
Crowding index ^c^	0.14 (0.01, 1.3)	0.08
Proportion of dwellings whose main water source is unprotected ^d^	0.17 (0.0, 11.7)	0.42
Proportion of dwellings that have a flush toilet	1.72 (0.5, 6.1)	0.40
Proportion of dwellings that use electricity for lighting	2.41 (0.5, 11.8)	0.28
Proportion of dwellings that own a radio	8.80 (0.0, 2449.2)	0.45
Proportion of dwellings that own an insecticide treated net	0.52 (0.02, 15.2)	0.71
Proportion of adult persons not literate in any language	0.11 (0.0, 13.6)	0.37
Presence of prison in the ward	4.38 (2.6, 7.3)	<0.001
Walking distance from QECH†	0.63 (0.3, 1.3)	0.22

* All but last covariate are from 2008 Census of Population and Housing [15]

† Shortest walking distance by mapped road from centre of ward to QECH

^a^ Non-grass roofing: tin/iron, tiles, asbestos, cement

^b^ Sealed floor: parquet, polished wood, vinyl, asphalt, ceramic tiles, cement, bricks

^c^ Crowding index: mean number or occupants divided by mean number of sleeping rooms

^d^ Unprotected water source: spring, river/stream, pond/lake, dam, rain water, unprotected well

### Ethics

All adults and adolescents presenting to the adult Accident & Emergency Department at QECH and fulfilling clinical indications had a blood culture collected through enhancement of routine clinical care provision. Persons aged 15 to 17 years attending hospital independently and cared for in adult wards are generally considered capable of providing their own consent for medical care under ethical norms in Malawi. Their location of residence was accessed retrospectively by interrogation of institutional database following ethical approval. For persons fulfilling enrolment criteria for a related clinical trial [1] additional data were collected after informed written consent was obtained from them if 18 years or older or from an attending parent or guardian if aged 15 to 17 years. We did not exclude populations at high risk for pneumococcal disease, including those on antiretroviral therapy or those residing in correctional institutions. This work was approved by the University of Malawi, College of Medicine Research Ethics Committee (approval numbers P.99/00/101 and P.01/08/609).

## Results

### Descriptive statistics

During 727 days of continuous surveillance, we recruited a cohort of 8,891 adults and adolescents aged 15 years or older who presented to QECH and fulfilled inclusion criteria. Among these participants we isolated a significant pathogen in 1724 (19.4%) including *S*. *pneumoniae* in 559 cases (6.3% of study cohort and 32.4% of pathogens), non-typhoidal Salmonella in 626 (7.0% and 36.3% of cohort and of pathogens respectively), *E*. *coli* in 177 (2.0% and 10.3% respectively), *C*. *neoformans* in 162 (1.8% and 9.4% respectively). Mean age of participants with pneumococcal bacteraemia and among those with negative culture was 34.3 (95% confidence interval (CI): 33.4, 35.1) and 34.2 (95%CI: 33.9, 34.4) years respectively, *P* = 0.86. Among the recruited bacteraemic cohort there were 46 cases that had *S*. *pneumoniae* isolated from cerebrospinal fluid. In addition a further 91 persons also had pneumococcal meningitis during the same period. Mean age was 31.9 (95%CI: 29.6, 34.3) years and 54 (60%) were female. Among bacteraemic patients the predominance of females in the cohort was associated with antiretroviral therapy (ART). Among persons on ART 61.8% were female, among those not on ART 56.5% were female (*P*<0.01).

### Incidence

**Fig 1:**
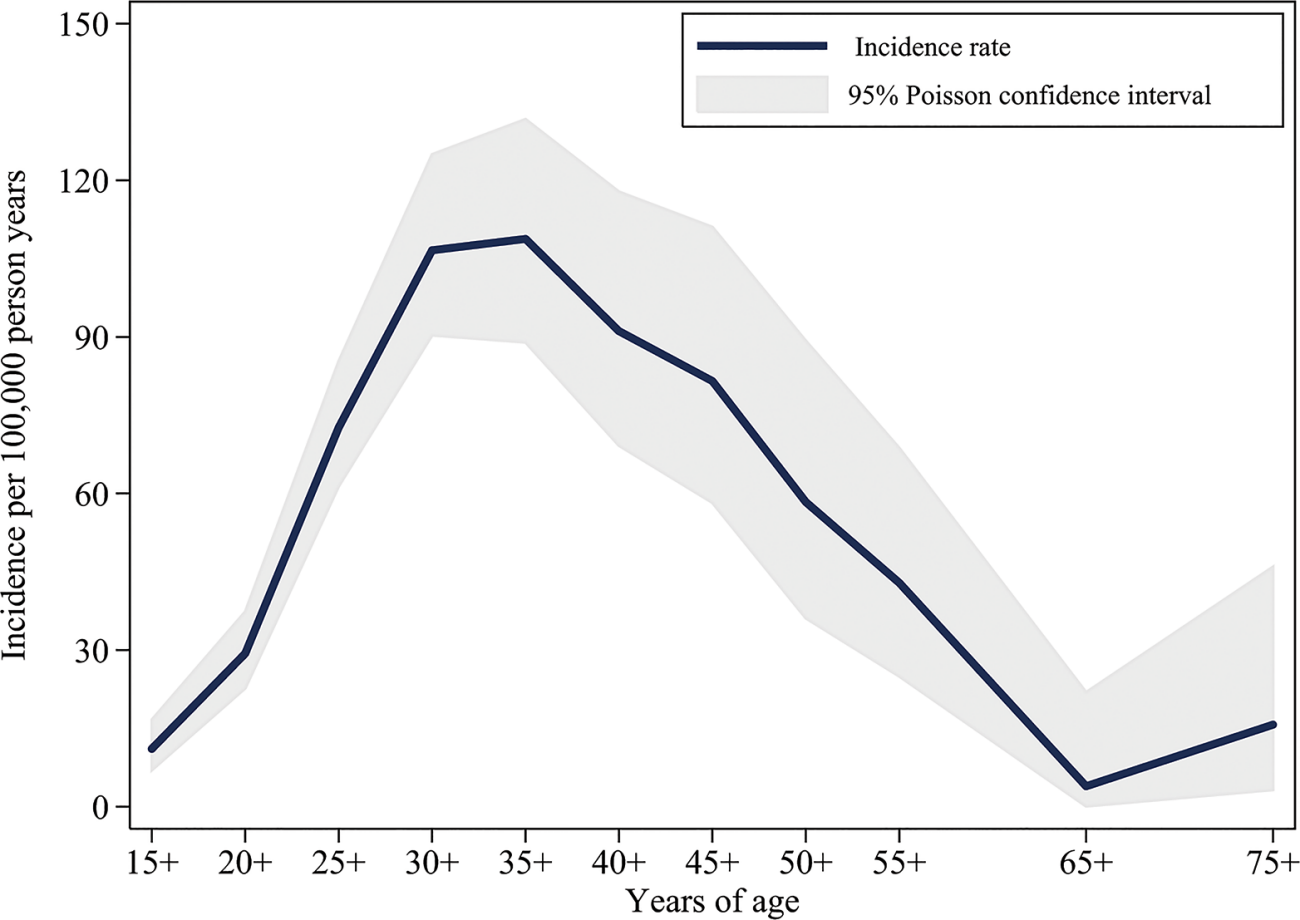
Population Incidence of Invasive Pneumococcal Disease in Blantyre, Malawi, among Adults ≥15 years, 1 January 2005 to 31 December 2006. Includes isolated pneumococcal bacteraemia, isolated pneumococcal meningitis and bacteraemic meningitis in Blantyre District (City and Rural).

Our surveillance spanned 1,119,158 person years of observation. Among adults 15 years and older resident in Blantyre district (city plus rural area) IPD incidence was 58.1 (95% CI: 89.0, 131.7) per 100,000 person years and peaking among 35 to 40 year olds at 108.8 (95%CI: 89.0, 131.7). For pneumococcal bacteraemia (not including non-bacteraemic meningitis) incidence was 50.0 (95%CI: 45.9, 54.3), and the peak at 35 to 40 year olds was 97.4 (95%CI: 78.7, 119.2). Because place of residence was known for bacteraemic cases we were able to calculate incidence within Blantyre city. In urban Blantyre incidence of pneumococcal bacteraemia was 60.6 (95%CI: 55.2, 66.5) and was greatest among 35 to 40 year olds at 114.8 (95%CI: 90.3, 143.9) (Fig 1).

**Fig 2:**
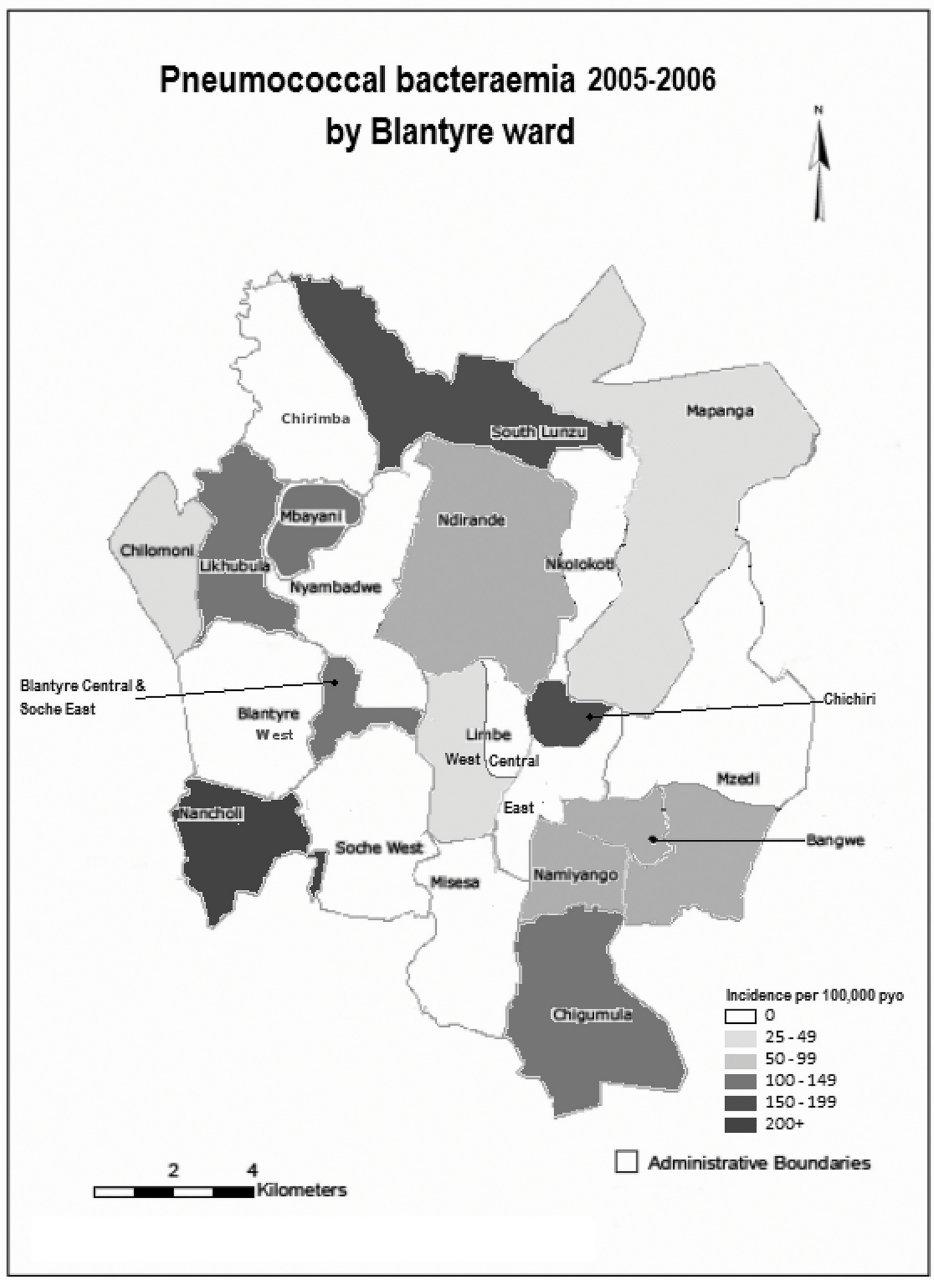
Map of Population Incidence of Pneumococcal Bacteraemia by Sub-district Ward among Adults ≥15 years, 1 January 2005 to 31 December 2006.

Incidence of bacteraemia varied among Blantyre wards and from many wards no cases were observed during the period of surveillance (Fig 2). Among wards where cases were observed, the mean incidence was 95.6 per 100,000 and the pattern of high incidence in mid-adulthood decades was consistent. For certain age groups in specific locations the incidence was very high (Fig 2). For example, Chichiri ward houses a large prison, 77 of the 80 (96.3%) prisoners in the cohort resided in Chichiri. Of 14 cases of pneumococcal bacteraemia from Chichiri ward, 12 (85.7%) were among prisoners. Including and excluding prisoners from case counts but assuming the same denominator, incidence in Chichiri was 187.1 (95%CI: 161.2, 215.8) per 100,000 and 26.7 (95%CI: 17.8, 39.3) per 100,000 respectively.

### Estimating under-ascertainment

Given the heterogeneity in ward level incidence, we calculated expected incidence by ward after adjusting for ward level covariates. Among all socio-demographic covariates, only the presence of a prison in the ward was associated with pneumococcal bacteraemia incidence (Incidence Rate Ratio 4.38, 95%CI: 2.63, 7.31, *P*<0.001) (Table 2), and none was associated with non-pneumococcal bacteraemia. There were no significant differences in any socio-demographic covariates between wards where cases were or were not detected (data not shown). Adjusting for socio-demographic covariates, the average predicted number of cases per ward was 43.7 and the mean incidence was 105.2 per 100,000. The expected total number of events adjusting for ward size was 1153 events, compared with 559 events actually observed, implying our sentinel site detected only 48.5% of the true disease burden. The socio-demographic adjusted model predictions had cases occurring in all wards, a phenomenon which we did not observe empirically (data not shown).

## Discussion

Malawi is largely rural but is undergoing rapid urbanisation[20,21]. Our study was located in a large urban and peri-urban setting typical of many population centres on the continent that have high HIV prevalence, poverty and dense urban living. In our setting pneumococcus is a leading cause of bloodstream infection and meningitis and is associated with substantial mortality. We set out to estimate the population incidence of invasive pneumococcal disease among adults living in Blantyre prior to the introduction of PCV13 into the infant vaccine schedule. We used data collected at the major hospital providing care to the residents of Blantyre, and used methods that have been carried forward into the infant vaccine era.

The minimum incidence rate for pneumococcal bacteraemia identified was 60.6 per 100,000 person years and close to double that in the age group with highest HIV prevalence. In some wards incidence was much higher and approached published rates for HIV infected adults[22]. Adults in Blantyre had a high incidence of pneumococcal bacteraemia compared with adult populations in other locations prior to conjugate vaccine introduction[23–26]. The profile of the incidence curve by age differs to that observed in low HIV prevalence settings, with peak incidence occurring in the middle decades of adulthood rather than in old age. The peak incidence for pneumococcal bacteraemia coincides with peak prevalence of HIV infection from large scale cross-sectional surveys in the same setting[20]. The association between HIV and high incidence of pneumococcal disease has been demonstrated clearly[26–28]. Comparison of incidence rates across populations with vastly differing HIV prevalence may be misleading. Age-distribution also differs for urban and rural location. Anecdotally many older persons return to their rural home villages upon leaving the workforce in the city.

We found heterogeneity in incidence within Blantyre district. Adjusting for socio-demographic covariates and for prison, the predicted incidence and expected number of events were much higher than was observed. Our modelling also predicted that cases should occur in all wards. The implication is that heterogeneity in incidence is at least in part due to under-ascertainment. However, it is likely that unmeasured covariates also accounted for the heterogeneity since the ward level socio-demographic covariates we were able to obtain were not independently associated with bacteraemia incidence and wards with no apparent cases did not differ socio-demographically from wards where cases were observed. Data on care seeking, on the functioning of health centres or performance indicators or referral patterns were not available, though these factors could also plausibly contribute to heterogeneity in incidence. Among the locations with high incidence one (Chichiri) was the site of a large prison while the other (Limbe West) housed a large police barracks. Closed communities are known to be at risk of pneumococcal disease outbreaks and in these instances one might also conjecture an association with HIV prevalence. Although in our cohort, being in prison was not associated with ART or TB therapy. Additionally, localised serotype specific outbreaks could plausibly occur in other local communal settings also contributing to the observed heterogeneity.

### Limitations

Our observed incidence rate is likely a minimal estimate. We did not use antigen detection or polymerase chain reaction, even in those previously receiving antibiotics, nor did we collect pleural fluid, all of which may have increased our yield. We focussed on invasive disease so have not accounted for the total burden of pneumococcal disease, such as non-bacteraemic pneumonia. We excluded from our numerator persons not usually resident in Blantyre district. The weak referral systems and suboptimal supply of drugs and services within Blantyre means we will have missed persons dying at home of pneumococcal disease. Despite this, our sentinel site represents the only publicly funded facility providing free care to the district. Although two private facilities exist, the associated costs make care prohibitively expensive for all but the very wealthiest. These facilities do not have bacterial culture capacity. The accuracy of our inter-censal population denominator estimates is unknown. We back-cast the population estimates in the study period using the method described above. Given the proximity of the study period to the subsequent census, we believe any error is likely to be small and will only make the incidence estimate conservative, we acknowledge that our method becomes less useful for longer term projections. Although our data do not immediately precede vaccine introduction, they do represent a period of stable clinical surveillance among adults in Blantyre and thereby provide a valid estimate of incidence during the study period. Despite Blantyre being typical of many rapidly urbanising population centres in sub-Saharan Africa, care should be taken in generalising our findings to rural areas of Malawi and indeed to other countries in the region. Our data contribute to the body of data from adult surveillance populations in Africa but should be taken in context of work from other sites such as the Gambia and South Africa, although adult data from the continent are still sparse [29,30]. It is likely however, that the challenges we faced in producing robust baseline estimates that would be useful for post-vaccine introduction comparisons would be similar in other poorly-resourced settings.

Our inability to find an association between ward level socio-demographic covariates and incidence may have to do with the arbitrary demarcation of wards. Economically disparate but adjacent populations that are counted within a single ward will mask any underlying socioeconomic differences. The socio-demographic indicators we used were collected in 2008. We did not attempt to infer the socio-demographic status for our study period 2005–6 so may have been misled by any rapid changes in living standards had these occurred. Additionally we lacked data on ward level HIV prevalence which may also contribute to heterogeneity in pneumococcal disease. The lack of health service performance data and referral patterns are important limitations, since these factors may account for heterogeneity in observed incidence at our referral centre. Since our model based predictions of expected incidence did not include these data, that estimate should be interpreted with caution. Despite these limitations we believe our argument stands—it is likely that our surveillance system, and so too those is similarly resource poor settings likely underestimates the true burden of disease. We plan to undertake an evaluation of primary health centre provision and performance, together with locally specific vaccine coverage rates among infants and HIV prevalence in households to inform future incidence evaluations and thereby integrate our surveillance systems with the district health office to further strengthen the health system of Blantyre. Additionally in order to infer indirect vaccine impact it is crucial to evaluate the dynamics of pneumococcal serotype distribution at population level before and after vaccine introduction, a process that is currently underway.

#### Implications for vaccine impact assessment

In much of Africa evaluation of vaccine impact will rely on before-after sentinel surveillance. Our finding of geographically heterogeneous ascertainment through differential care seeking or survivorship bias has important implications for the design and conduct of post vaccine introduction surveillance in a complex urban and peri-urban setting such as Blantyre. Active surveillance systems that reduce ascertainment bias may find a paradoxical increase in disease if relying solely on historical comparisons. Our finding of high pneumococcal incidence in age groups that mirror HIV prevalence together with local hot spots also have implications for the post-vaccine era, since population reservoirs of pneumococcal carriage and transmission in adults and other age groups ineligible for vaccination may persist following vaccine introduction in infants. Malawi introduced the pneumococcal conjugate vaccine to the child population in November 2011. Surveillance is continuing to observe possible reductions in incidence of adult disease and studies examining indirect effectiveness of vaccination on older children and adults are also under way. Surveillance in the post vaccine era will have to take into account heterogeneity in disease and ascertainment if accurate estimates of vaccine direct and indirect effectiveness are to be made.
